# Effects of plyometric-based structured game active breaks on fundamental movement skills, muscular fitness, self-perception, and actual behaviour in primary school students

**DOI:** 10.5114/biolsport.2024.132991

**Published:** 2023-12-21

**Authors:** Andrew Sortwell, Kate O’Brien, Aron Murphy, Rodrigo Ramirez-Campillo, Benjamin Piggott, Gregory Hine, Michael Newton

**Affiliations:** 1 School of Health Sciences and Physiotherapy, University of Notre Dame Australia, Sydney, Australia; 2 Education and Research Directorate, Sydney Catholic Schools, Sydney, Australia; 3 Exercise and Rehabilitation Sciences Institute, School of Physical Therapy, Faculty of Rehabilitation Sciences, Universidad Andres Bello, Santiago, Chile; 4 School of Health Sciences and Physiotherapy, University of Notre Dame Australia, Perth, Australia; 5 School of Education, University of Notre Dame Australia, Perth, Australia

**Keywords:** Children, Motor skills, Resistance training, Self-concept, School-setting, Learning

## Abstract

This study examined the effects of plyometric-based structured game active breaks on fundamental movement skills (FMS), muscular fitness, student self-perception, and teacher’s rating of actual behaviour in Grade 3 and 4 students. Primary school children aged 8–10 years old, from four classes, were cluster-randomly assigned to an intervention group (IG) (*n* = 54) or a control group (CG) (*n* = 48). The IG participated in structured plyometric-based game active breaks for 7–10 minutes daily, for six consecutive weeks. The CG resumed their regular daily school routine. FMS were assessed with the Canadian Agility Movement Skills Assessment test, and muscular fitness with the standing long jump (SLJ), countermovement jump (CMJ), and seated medicine ball chest throw tests. The Self-Perception Profile for Children and the Teacher’s Rating Scale of Child’s Actual Behaviour assessed student self-perception and teacher’s perception of student actual behaviour, respectively. A significant (*p* < 0.01) interaction group by time was observed, with greater improvements in the IG compared to the CG in FMS (%diff = 13.11, ƞp^2^ = 0.12), SLJ (%diff = 6.67, ƞp^2^ = 0.02), seated medicine ball chest throw (%diff = 4.69, ƞp^2^ = 0.08), student social self-perception (%diff = 9.31, ƞp^2^ = 0.10), student scholastic self-perception (%diff = 7.27, ƞp^2^ = 0.10), and teacher perception of student social competence (%diff = 8.31, ƞp^2^ = 0.05). No difference (*p* > 0.05) was found in other variables. Integrating plyometric-based structured game active breaks into primary school settings evidenced improvement in FMS, muscular fitness, student self-perception, and teacher’s rating of student actual behaviour.

## INTRODUCTION

Schools are continually seeking strategies to improve student learning and enhance wellbeing. A contemporary strategy gaining attention is short physical activity breaks integrated into the lesson, primarily aiming to improve cognition, executive functions, and learning outcomes [1]. Typically, physical activity breaks, or active breaks, consist of 5–10 minutes of physical activity and are integrated into classroom time after 40–60 minutes of learning [1]. Previous studies have demonstrated that embedding an active break into the school day can also increase children’s cognitive performance [2], and physical activity levels [1]. However, there has been little examination of the effect on student fundamental movement skills (FMS), muscular fitness, student self-perception (i.e., global self-worth, scholastic, athletic and social competence) and student actual behaviour [1].

Over the last 10 years, schools implementing active breaks have tended to use the Finnish model, characterised by unstructured physical activity or play, contributing to healthier children and improved classroom learning [3]. Unstructured breaks have been suggested to result in positive changes in the critical region of the brain’s executive control centre, the prefrontal cortex, which is responsible for problem-solving, decision-making, planning, and regulating emotions, and may result in improved student behaviour and engagement [4]. However, as noted in the meta-analysis by Infantes-Paniagua et al. [1], one challenging aspect of unstructured activity breaks is ensuring that all students are active and moving sufficiently to benefit from the intended outcomes.

As knowledge regarding child development and learning has grown, the need to shape more effective educational practices within the set learning time has also increased. By successfully integrating in-sights from sports science, neurosciences, psychology, sociology, and learning sciences, more effective holistic approaches are likely to emerge in education. Therefore, a deeply integrated approach to classroom practices (i.e., active breaks) with multifaced benefits needs further examination. For example, there appears to be no studies examining active breaks that have been designed with the additional purpose of improving students’ psychomotor domain of learning. This area warrants further investigation, considering that executive function seems especially sensitive to coordinative complex movements [5]. Moreover, FMS proficiency is associated with scholastic competence, social acceptance, global self-worth and athletic competence [6, 7]. This association is significant since positive self-perceived competence is associated with resilience, enhanced motivation, and reduced anxiety [8]. Therefore, research is needed to examine a multi-purpose approach to active breaks that may result in a broader range of benefits for students, potentially improving FMS and related aspects of student perceived competence, hence a more effective use of active break time within the school setting.

Plyometric exercises are broadly used in sports training to improve physical performance by enhancing the use of the stretch-shortening cycle, in which a lengthening movement (eccentric) is quickly followed by a shortening movement (concentric) and to optimise related neuro-mechanical mechanisms [9]. In primary-aged students, it has been demonstrated that short-term plyometric training regimens can improve motor coordination, FMS, and muscular fitness [10, 11]. Embedding plyometric exercises into structured games during active breaks could be an innovative approach that may lead to multifaceted benefits for students.

Using a structured game approach to active breaks rather than unstructured physical activity, could also provide students with an improvement in self-perception by promoting both physical and psychological advantages [12, 13]. Due to the social nature of structured collaborative games, participation provides opportunities for social interaction and companionship, and may have more significant benefits for social and mental wellbeing than unstructured play [12, 13]. Therefore, through interaction with peers during structured plyometric-based active breaks, children may be more likely to experience physical, psychological, social benefits, and improved classroom behaviour conduct outcomes [1].

Therefore, the aim of this study was to determine the effects of plyometric-based structured game active breaks on FMS, muscular fitness, student self-perception, and actual behaviour in Grade 3 and 4 students. It was hypothesised that plyometric-based structured game active breaks would improve FMS competence, muscular fitness parameters, student self-perception, and student’s actual behaviour.

## MATERIALS AND METHODS

### Participants

The sample comprised of 102 primary school children, 55 females and 47 males, aged 8 to 10 years old, from two Grade 3 and two Grade 4 classes at a primary school, who volunteered for this study. Inclusion criteria were: students aged 8 to 10 years, in Grades 3 or 4 and without musculoskeletal injuries. Using G*Power software (Heinrich-Heine-Universität, Düsseldorf, Germany) with P value and power fixed at 0.05 and 90%, respectively, with medium effect size, a sample size of 44 students per group was required to detect any interaction effect. After the study protocol received approval from the Ethics Committee of the University of Notre Dame Australia, and prior to familiarisation sessions and any data collection sessions, all teachers, students and their parents or guardians received a written explanation of all study procedures, were offered the opportunity to attend information sessions, and allowed to ask questions of the research team. Subsequently, teachers, students and their parents agreed to participate in the study with informed signed consent. Parents/guardians with their child completed a physical activity readiness questionnaire to ensure they were physically healthy to participate in this study. The parents/guardians also completed the Children’s Leisure Activities Study Survey (Class) parental proxy questionnaire according to their child’s physical activities during a typical week [14] (Table 1). All participants in the study were informed that they could withdraw from the study at any time without penalty. All study procedures conformed to the 2013 Declaration of Helsinki guidelines.

**TABLE 1:** Characteristics of the two groups

Characteristics	IG (n = 54)	CG (n = 48)	*p-value*
Gender (girls/ boys)	55.6/ 44.4	52.1/ 47.9	0.73
Age (years)	8.96 ± 0.69	9.13 ± 0.64	0.23
Height (cm)	138.26 ± 6.69	138.15 ± 6.04	0.93
Weight (kg)	33.94 ± 6.50	34.41 ± 8.62	0.76
BMI (kg/m^2^)	17.67 ± 2.62	17.89 ± 3.71	0.73
Physical activity level (h/wk)	10.90 ± 4.93	11.42 ± 6.37	0.16

Notes: Data are reported as mean ± SD or percentage, and level of significance (p-value) of the comparison between groups. P-values were calculated using an unpaired t-test. Abbreviations: IG: intervention group; CG: control group; BMI: body mass index; h/wk: hours per week. *: Statistically significant with p < 0.05 comparison between groups.

### Experimental design

A cluster-randomised controlled trial was conducted using four natural classes in a primary school. The four classes were assigned randomly to the following study groups: intervention group (IG) (*n* = 54) or control group (CG) (*n* = 48). The participants’ general characteristics are outlined in Table 1. The exclusion criteria for data were: not attending 85% of the plyometric-based structured game breaks, or missing more than three game breaks consecutively.

### Procedures

All procedures were performed during the final school term for the year (October, November, and December 2022). Prior to the commencement of the research study, students were familiarised on the assessment protocols over the duration of a school day. At the end of the familiarisation sessions, all participants were instructed to refrain from strenuous physical activity or sport the day before testing. Pre- and post-test measurements were conducted at the same time of day for each class to control for potential diurnal variation in test results by completing post-tests within one hour of the pre-tests. The students completed the tests in the same student sequence and test order on the first and second day in the pre- and post-testing weeks using identical equipment, positioning, and technique. To eliminate the effects of the environment and weather conditions (e.g., direct sunlight, wind, temperature) on performance, the pre and post-tests on the first day of testing were performed in the school hall and then on the second day in an undercover shielded area. The research assistants were trained and familiar with the tests they administered; they were also blinded to the groups to which students belonged. The physical assessment tests were conducted after a general warm-up of jogging, running, lower-body callisthenics, and whole-body dynamic stretches. For each test described in the following sections, all participants had a brief familiarisation warm-up before executing the tests.

### Anthropometric measurements

Anthropometric measurements were obtained using a mobile stadiometer (SECA 217, Hamburg, Germany) and an electronic scale (SECA 803, Hamburg, Germany) during pre- and post-test sessions following the International Society for the Advancement of Kinan-thropometry (ISAK) guidelines. The body height (± 0.1 cm) was measured with the participant standing barefoot using a stadiometer. Body mass (± 0.1 kg) was measured using the electronic scale, with the participants standing barefoot, with feet together. Body mass index (BMI) was calculated as weight [kg]/ (height [m])^2^.

### Canadian agility and movement skill assessment

The Canadian Agility Movement Skill Assessment, a valid and reliable measure to evaluate children’s FMS competence [15], was implemented following the protocol described by Longmuir et al. [16]. The test requires the students to complete a prescribed movement course involving a sequence of movement skills: two-foot jumping, sliding, catching, throwing at a target, skipping, one-foot hopping, and kicking a soccer ball through cones. A skill score based on a criterion-references assessment is determined, and the overall time to complete the movement course is recorded. The overall raw score was quantified as a sum of the skill and time criteria scores. The students were given two practice trials followed by two timed and scored attempts. All attempts were recorded and the best score was used for analysis.

### Standing long jump test

The standing long jump test (SLJ) is a reliable and valid measure [17], and was used to assess maximum horizontal jump distance according to the previously described protocol [17]. The jump distance was measured using two parallel 10-metre fibreglass metric tapes 40 centimetres apart, from the marked starting line to where the heel contacted the ground on landing. The test was performed three times after an initial trial jump, separated by one-minute rest. All jumps were recorded and the furthest jump result was used for analysis.

### Countermovement jump

A countermovement jump (CMJ) was used to assess lower-body muscular power: as the test is reliable and valid [17]. The student’s maximum CMJ height was measured using a Vertec (Yardstick, Swift Performance Equipment, Australia) and according to the previously described protocol [18]. Students had four attempts, the first being a practice and then the subsequent three attempts recorded. The CMJ height was calculated by subtracting the standing reach height from the jump height, and then the best maximum jump height was used for subsequent analysis.

### Seated medicine ball chest throw

The seated medicine ball chest throw test, a reliable and valid measure of upper body muscular power in children [19], was used to assess upper body muscular power according to the described protocol of Faigenbaum et al. [20]. The test was performed using a 20 cm diameter 1 kg medicine ball powdered with magnesium carbonate, two 10-metre fibreglass metric tapes one metre apart, affixed with clear tape to a black rubber floor mat. The test was performed three times after an initial trial chest throw, separated by one-minute rest, with all test throws recorded and the furthest throw distance used for analysis.

### Self-perception profile for children questionnaire

The Self-Perception Profile for Children (SPPC) questionnaire was administered to students to evaluate changes in student perceived competence [21]. The SPPC is a validated and reliable psychometric measure in children [22]. The questionnaire consists of six questions per the following subscales: athletic competence, social competence, scholastic competence, and global self-worth. Each question is answered on a four-point scale, with higher scores being a more positive self-perception. The questionnaire was administered to students in the classroom environment by a trained research assistant, with the teacher present, according to the protocol described by Harter [21].

### Teacher’s rating scale of child’s actual behaviour questionnaire

The Teacher’s Rating Scale of Child’s Actual Behaviour questionnaire [21], was used to assess changes in each student’s domain of behaviour conduct and athletic, social and scholastic competence based on teacher observations. The questionnaire has good internal reliability, internal consistency, test–retest reliability and construct and convergent validity [22]. The questionnaire contained twelve items scored on a four-point scale. Teachers were directed to complete the questionnaire for each student according to the protocol of Harter [21] and rate the student’s actual behaviour in each domain.

### Intervention program

The six-week intervention consisted of plyometric-based structured game active breaks in the middle of a two-hour learning block in the class’s daily routine (see Table 2). Based on previous research, the plyometric-based games were specifically designed for primary school children [23, 24]. The intervention was performed each school day on school premises for seven to ten minutes. At the start of each game break, the classroom teacher, who had received training in delivering the games, demonstrated the proper game technique and reviewed procedures. The intervention included ten games that utilised plyometric exercises (jumping, skipping, hopping, medicine ball throw downs, rapid passing) using body weight and medicine balls (2 kg), with students playing the games in small groups of five to six. The six-week program was divided into two phases, each consisting of three weeks, with the second phase being of higher intensity by using more complex movements such as single-leg hops instead of two-legged jumps to maximise adaptations [25]. A fidelity checklist and logbook were used each day to monitor implementation for adherence and quality. The fidelity checklist and logbook included key aspects of the intervention, such as teacher delivery of the scheduled game, student participation, start and finish times and any occurrence of injuries. The lead researcher and an independent observer directly observed 96% of the implemented plyometric-based structured game active breaks for treatment integrity, demonstrating 100% adherence.

**TABLE 2:** Overview of the plyometric-based structured games

Name	Overview	Sets
1. Kangaroo Ninja Warrior Relay	Jump 10 m with MB. Five double-handed MB throwdowns. Skip 10 m to starting position with MB.	4

2. Skip and Pick Up Bean Bags	Skip 10 m, pick up the bean bag, and turn around. Skip to starting position.	5

3. Throw Down and Travel Around the Globe	Three double-handed throwdowns of the MB. Then passed rapidly.	6

4. Bean Bag Pickup	Four two-legged rapid jumps in and out of hoops, pick up one bean bag and then turn around. Four two-legged rapid jumps back to starting position.	5

5. Basketball Hot Seat	In a circle formation, catch the basketball and rapidly pass it back to the person in the circle’s centre. Each student has a turn in the center.	4

6. Ultimate Ninja Warrior Relay	One-legged hopping along a 10 m distance with MB. Five single-handed MB throwdowns. Skip 10 m back to starting position with MB.	4

7. Sticky Knees Relay	Jump with a latex ball between the knees for 4 m, then turn around. Jump back with the latex ball between the knees back to starting position.	4

8. Single Hand Throw Down and Travel Around the Globe	With the group in a circle formation, perform three single-handed throwdowns with MB. Then rapidly pass to the next student.	6

9. Single Leg Bean Bag Pickup	Four single leg hops in and out of hoops, pick up one bean bag and then turn around. Four single leg hops back to starting position.	5

10. Basketball Hopping Hot Seat	In a circle formation, hop on the spot, catch the basketball, and rapidly pass back to the person in the circle’s center. Each student has a turn in the center. Continual hopping on either leg.	4

Note: The shaded area denotes the second phase of the program with more complex movements. All jumps were performed horizontally. MB: 2-kilogram medicine ball.

### Statistical Analysis

All statistical analyses were performed using the IBM SPSS Statistics for Windows software (Version 28.1; IBM Corp., Armonk, NY). Descriptive data were calculated for all variables. The normality of variables was tested using the Shapiro-Wilk test procedure. Descriptive variables from both groups and baseline data were compared using an unpaired t-test. The reliability of each test involving trials was assessed by calculating the intra-class correlation coefficient (ICC) [26]. A two-way ANOVA with repeated measures was used to determine the effects of the group (IG and CG) and time (baseline and post-intervention) as the between-and within-subject factors in the dependent variables. Effect size (ES) was evaluated with eta partial squared (ƞp^^2^^). According to Hopkins guidelines, the effect size (eta-squared; η^2^) was established as follows: 0.01 – small, 0.06 – medium, and 0.14 – large [27]. To control for Type 1 errors, a Bonferroni post-hoc test was conducted to identify where significant differences occurred and to determine the within-group changes. Within effect was assessed using Cohen’s *d* and interpreted as trivial (< 0.20); small (0.20–0.59); moderate (0.60–1.19); large (1.20–1.99); and very large (> 2.00) [27].

## RESULTS

There were no significant between-group differences (*p* > 0.05) in the descriptive variables and baseline data (see Tables 1 and 3). The ICC of all physical assessment tests ranged between 0.813 and 0.983 (*p* < 0.001). For six tests, statistically significant improvements (*p* < 0.05) were observed in the intervention group compared to the control group. The pre- and post-test outcomes for both groups and the statistical analysis results are displayed in Tables 4 and 5.

**TABLE 3:** Baseline data and differences between groups

Characteristics	IG (n = 54)	CG (n = 48)	*p-value*
Canadian agility movement skill assessment (score)	19.22 ± 3.90	19.00 ± 4.15	0.39
CMJ (cm)	27.39 ± 5.49	27.94 ± 5.72	0.31
Seated medicine ball chest throw (m)	2.77 ± 0.50	2.73 ± 0.46	0.33
SLJ (m)	1.35 ± 0.20	1.34 ± 0.24	0.71
Self-perception of athletic competence (score)	18.41 ± 3.96	18.63 ± 4.12	0.39
Self-perception of global self-worth (score)	19.28 ± 3.39	19.23 ± 3.54	0.47
Self-perception of scholastic competence (score)	17.19 ± 3.34	16.42 ± 4.56	0.17
Self-perception of social competence (score)	16.98 ± 3.60	17.10 ± 4.28	0.44
Teacher rating of athletic competence (score)	9.28 ± 2.26	8.71 ± 2.68	0.19
Teacher rating of behavioural conduct (score)	11.04 ± 2.02	10.83 ± 2.36	0.68
Teacher rating of scholastic competence (score)	9.13 ± 2.72	8.98 ± 3.33	0.23
Teacher rating of social competence (score)	8.02 ± 2.41	9.15 ± 2.96	0.14

Note: Data are reported as mean ± SD, and level of significance (p-value) of the comparison between groups. P-values were calculated using an unpaired t-test. Abbreviations: IG: intervention group; CG: control group; CMJ: countermovement jump; SLJ: standing long jump. *: Statistically significant with p < 0.05 comparison between groups.

**TABLE 4:** Changes in selected physical indicators from pre- to post-test

Group	Pre-test	Post-test	Δ%	ANOVA (group × time)	Bonferroni post-hoc (time) p-values (ES: *d*)
**Fundamental Movement Skills**					

Canadian agility movement skill assessment (score)					
IG	19.22 ± 3.90	21.74^*^ ± 3.59	**13.11**	***F*_(1,100)_ = 14.09,**	**< 0.001** (0.81)
CG	19.00 ± 4.15	19.44 ± 3.76	1.02	***p* < 0.001, ƞp^2^ = 0.12**	0.28 (0.18)

**Muscular Fitness**					

CMJ (cm)					
IG	27.39 ± 5.49	29.33 ± 5.71	**7.08**	*F*_(1,100)_ = 1.90,	**< 0.001** (0.35)
CG	27.94 ± 5.72	28.89 ± 6.95	3.40	*p* < 0.17, ƞp^2^ = 0.02	0.07 (0.16)

Seated medicine ball chest throw (m)					
IG	2.77 ± 0.50	2.90^*^ ± 0.52	**4.69**	***F*_(1,100)_ = 9.70,**	**< 0.001** (0.25)
CG	2.73 ± 0.46	2.72 ± 0.47	-1.00	***p* < 0.002, ƞp^2^ = 0.08**	0.90 (0.008)

SLJ (m)					
IG	1.35 ± 0.20	1.44^*^ ± 0.21	**6.67**	***F*_(1,100)_ = 19.58,**	**< 0.001** (0.41)
CG	1.34 ± 0.24	1.35 ± 0.26	0.07	***p* < 0.001, ƞp^2^ = 0.16**	0.35 (0.04)

Note: Values are presented as mean ± SD and percentage of variation from baseline to follow-up (Δ%); F values, level of significance (*p*-value), and effect sizes as eta partial squared (ƞp^2^) following two-way repeated measures ANOVA; post-hoc multiple comparison analyses with Bonferroni adjustment; Cohen’s *d* (*d*) in brackets of the within groups comparison are also displayed.

* : Statistically significant difference between intervention and control group in post-intervention values. Numbers in bold represent statistically significant differences (*p* < 0.05). IG: intervention group (plyometric-based structured game active break group); CG: control group; CMJ: countermovement jump; SLJ: standing long jump.

**TABLE 5:** Change in self-perception and child’s actual behaviour from pre- to post-test

Group	Pre-test	Post-test	Δ%	ANOVA (group × time)	Bonferroni post-hoc (time) p-values (ES: *d*)
**Student Self-Perception**					

Athletic competence (score)					
IG	18.41 ± 3.96	18.85 ± 3.77	2.39	*F*_(1,100)_ = 1.26,	0.34 (0.12)
CG	18.63 ± 4.12	18.31 ± 4.46	1.02	*p* = 0.264, ƞp^2^ = 0.01	0.53 (0.07)

Global self-worth (score)					
IG	19.28 ± 3.39	20.21 ± 3.24	**4.82**	*F*_(1,100)_ = 0.06,	**0.049** (0.28)
CG	19.23 ± 3.54	20.25 ± 3.60	**5.30**	*p* = 0.81, ƞp^2^ = 0.001	**0.03** (0.29)

Scholastic competence (score)					
IG	17.19 ± 3.34	18.44^*^ ± 3.41	**7.27**	***F*_(1,100)_ = 11.30,**	**< 0.001** (0.37)
CG	16.42 ± 4.56	16.06 ± 4.46	-2.24	***p* < 0.001, ƞp^2^ = 0.10**	0.31 (0.08)

Social competence (score)					
IG	16.98 ± 3.60	18.56^*^ ± 3.04	**9.31**	***F*_(1,100)_ = 11.30,**	**< 0.001** (0.47)
CG	17.10 ± 4.28	17.04 ± 4.31	-1.00	***p* < 0.001, ƞp^2^ = 0.10**	0.86 (0.015)

**Teacher’s Rating Scale of Child’s Actual Behaviour**					

Athletic competence (score)					
IG	9.28 ± 2.26	10.10 ± 1.99	8.84	*F*_(1,100)_ = 1.66,	0.20 (0.38)
CG	8.71 ± 2.68	9.15 ± 2.63	5.10	*p* = 0.201, ƞp^2^ = 0.016	0.21 (0.17)

Behavioural conduct (score)					
IG	11.04 ± 2.02	11.26 ± 1.63	1.99	*F*_(1,100)_ = 0.003,	0.18 (0.11)
CG	10.83 ± 2.36	11.04 ± 1.97	1.93	*p* = 0.954, ƞp^2^ = 0.00	0.24 (0.09)

Scholastic competence (score)					
IG	9.13 ± 2.72	9.48 ± 2.51	3.83	*F*_(1,100)_ = 1.22,	0.07 (0.12)
CG	8.98 ± 3.33	9.02 ± 3.13	0.04	*p* = 0.272, ƞp^2^ = 0.01	0.84 (0.07)

Social competence (score)					
IG	8.02 ± 2.41	8.72^*^ ± 2.43	**8.73**	***F*_(1,100)_ = 4.715,**	**< 0.001** (0.29)
CG	9.15 ± 2.96	9.27 ± 2.91	1.31	***p* < 0.05, ƞp^2^ = 0.05**	0.52 (0.04)

Note: Values are presented as mean ± SD and percentage of variation from baseline to follow-up (Δ%); F values, level of significance (*p*-value), and effect sizes as eta partial squared (ƞp^2^) following two-way repeated measures ANOVA; post-hoc multiple comparison analyses with Bonferroni adjustment and Cohen’s *d* (*d*) in brackets of the within groups comparison are also displayed.

* : Statistically significant difference between intervention and control group in post-intervention values. Numbers in bold represent statistically significant differences (*p* < 0.05). IG: intervention group (plyometric-based structured game active break group); CG: control group.

## DISCUSSION

This study aimed to evaluate the changes in FMS competence, muscular fitness, self-perceived competence, and teacher rating of student actual behaviour resulting from the effects of engaging in a daily active break consisting of plyometric-based structured games nested in the middle of a 2-hour block of curriculum time in primary school children. The results indicate that the IG group significantly improved on the Canadian Agility Movement Skill Assessment, SLJ, seated medicine ball chest throw, self-perceived social and scholastic competence, and teacher rating of social competence compared to the CG that continued with the regular daily school routine. There was no significant difference between the groups for CMJ, self-perceived athletic competence and global self-worth, teacher rating of student athletic competence; student behaviour conduct; and scholastic competence. No injuries occurred throughout the study, and observations suggest that plyometric-based structured games led by general classroom teachers are a safe approach for Grades 3 and 4 students. As such, the results of the current study are novel and provide evidence of a multitude of benefits for students performing plyometric-based structured game active breaks, in contrast to the limited cognitive outcomes currently advocated for active breaks.

Student FMS proficiency is an important prelude to children’s engagement in physical activity and sports [28]. In the current study, FMS was improved by 13.1% (medium ES) in the IG after six weeks of structured plyometric-based breaks. The results of the current study are consistent with those of Sortwell et al. [11], which showed that plyometric circuit of exercises within the primary school setting positively affects FMS competence (12.91%; ES = 0.60, large) in children aged 7–8 years. The current results could be expected, given that the structured games included plyometric exercises that have been shown to enhance FMS in primary-aged students [10, 23], and improve motor performance in young athletes [9]. Furthermore, the plyometric horizontal jumps used in the current study have also been suggested to be an efficient method for enhancing multi-vector performance for sport, hence the potential transference to the improved performance in the Canadian Agility Movement Skills Assessment test [29]. Even though in the current study the plyometric-based structured games were performed for only 7 to 10 minutes, the weekly frequency of the games was high (i.e., daily) and produced similar FMS enhancements to other studies that have engaged young school children in plyometric training [10, 23]. The improvement in FMS is most likely due to muscle adaptation driven by the plyometric exercises, resulting in possible neural and structural enhancements [9]. Given that FMS competence is generally undeveloped in children [28], embedding plyometrics-based structured games into school active breaks appears to be an efficient and effective method to improve FMS in children.

Our results indicate that the plyometric-based structured game active break intervention effectively improved muscular fitness, as the SLJ significantly increased by 6.7% (large ES), and the seated medicine ball chest throw increased significantly by 4.7% (medium ES). These improvements align with the results of previous studies involving primary school-aged children [10, 23, 24]. Even though the IG improved CMJ by 7.1%, this did not reach a significant difference between the groups. This is most likely due to the affinity between the type of jumps (i.e., horizontal) used in the games and the SLJ test [29]. Improvements in the SLJ and seated medicine ball chest throw may also be more related to biomechanical parameters such as maximal isometric voluntary force, changes in the stiffness of various elastic components of the muscle-tendon complex, musculoskeletal contractile properties, and rate of force development in the muscles in the children [30]. These findings have significant practical relevance for primary school students, since muscular fitness is associated with health and skills-related fitness components that support daily living, participation in physical activities and sports, and improvements in adiposity and cardiovascular disease risk factors [31].

The current study lends credence to previous research that reports on interventions emphasising the development of motor skills (i.e., FMS) and being associated with a boost in perceived scholastic competence [32]. Evidence suggests that structured exercise with-in the school setting can improve dimensions of cognitive function, including cognitive planning and reaction time in students [33]. More-over, an extensive body of research indicates that fitness exercise can improve the scholastic performance of children via molecular, cellular, and behavioural brain mechanisms [34]. Therefore, it is plausible that students’ perceived scholastic competence might have improved following the enhancement of FMS competence, resulting from perceptual-motor behaviours facilitating readiness to learn, thus, scholastic performance [35]. Furthermore, research in preschool children has found a strong relationship between complex fitness components such as hopping on one leg (as used in the current study) and cognitive attention [32], reinforcing the benefit of using plyometrics within active breaks. Despite the student improvement in self-perception in the scholastic competence domain, only minimal non-significant improvements (3.8%) in teacher perception of students scholastically were found.

Significantly improved student self-perceived social competence and teacher-rating of student social competence indicates the potential for plyometric-based structured game active breaks to contribute to enhanced student social competence and interpersonal skills. These findings are similar to the work by Kao [36], which engaged students in activities that enhance movement skills, and psychomotor development through structured sports and games that can promote teamwork, sharing, which led to enhanced interpersonal relationships with peers. Furthermore, using a structured game approach to active breaks helps negate students experiencing negative self-perception as a result of social exclusion, which can occur in unstructured play and physical activity [37]. Therefore, the delivery of plyometric-based structured game active breaks rather than only free-play breaks may be a more effective strategy to facilitate inclusion and build student self-confidence [38]. Previous literature also supports this finding emphasising the benefits of using fitness activities and movement experiences in schools that are structured [39]. The outcomes of the current study corroborate the notion that forming student relationships through plyometric-based structured game active breaks may effectively improve social competence.

The intervention induced no significant differences in student athletic competence, global self-worth and teacher perception of student athletic competence and behaviour conduct. The absence of a between-group effect for measures of athletic competence in the current study is in accordance with the results of longitudinal studies, indicating that perceived athletic competence in children is relatively constant over time [40]. The minimal change in the general classroom teacher’s rating of student athletic competence may also be due to the teacher being absent in the physical education lessons to view the translation into sports, since a specialist physical education teacher leads the lessons. While research indicates that high levels of athletic competence are positively associated with greater global self-worth [41], these relationships can be mediated to a substantial extent by BMI and cardiovascular fitness [42]. Similar to studies that have used classroom-based physical activity, participation did not result in a chronic effect on student behaviour conduct [43, 44]. Future studies should investigate the relationship between structured active breaks and the acute effect on student behaviour conduct and explore the effects of changing the current intervention parameters, such as increasing the duration from six weeks and altering the movement intensity, and assessing the effect on athletic competence, global self-worth, and behaviour conduct.

In the current study, we acknowledge some potential limitations. Firstly, we involved only students in Grades 3 and 4. Future studies should confirm its replicability in younger and older students. Furthermore, the research study was short-term, and the results do not provide insight into long-term changes or adaptations.

## CONCLUSIONS

This is the first study that has investigated the effects of using plyometric-based structured games during primary school active breaks on FMS, muscular fitness, student self-perception and actual behaviour. The results of this study demonstrate the efficacy of using plyometric-based structured games during daily active breaks in the primary school setting and suggest that within six weeks, positive changes are produced in FMS, measures of muscular fitness (SLJ and seated medicine ball chest throw) and student social and scholastic competence. Overall, this study illuminates diverse student benefits derived from plyometric-based structured activities during primary school active breaks, surpassing the commonly emphasised academic-related cognitive outcomes and contributing to existing knowledge of active breaks by providing a new innovative approach that teachers can deliver. The evidence presented in this study may also help school practitioners design active break activities that help the student reset their focus for continual learning of the set curriculum and achieve psychomotor and psychological benefits that support the student’s holistic development.
