# Erratum to: Effects of plyometric-based structured game active breaks on fundamental movement skills, muscular fitness, self-perception, and actual behaviour in primary school students

**DOI:** 10.5114/biolsport.2024.142165

**Published:** 2024-09-05

**Authors:** Andrew Sortwell, Kate O’Brien, Aron Murphy, Rodrigo Ramirez-Campillo, Benjamin Piggott, Hine Gregory, Newton Michael

**Affiliations:** 1 School of Health Sciences and Physiotherapy, University of Notre Dame Australia, Sydney, Australia; 2 Education and Research Directorate, Sydney Catholic Schools, Sydney, Australia; 3 Exercise and Rehabilitation Sciences Institute, School of Physical Therapy, Faculty of Rehabilitation Sciences, Universidad Andres Bello, Santiago, Chile; 4 School of Health Sciences and Physiotherapy, University of Notre Dame Australia, Perth, Australia; 5 School of Education, University of Notre Dame Australia, Perth, Australia

**Keywords:** Children, Motor skills, Resistance training, Self-concept, School-setting, Learning

The authors would like to correct the error in the publication of the original article with this erratum for your reading.

**Erratum notice**:

In the previously published version of the abstract, the following text was presented:

“This study examined the effects of plyometric-based structured game active breaks on fundamental movement skills (FMS), muscular fitness, student self-perception, and teacher’s rating of actual behaviour in Grade 3 and 4 students. Primary school children aged 8–10 years old, from four classes, were cluster-randomly assigned to an intervention group (IG) (*n* = 54) or a control group (CG) (*n* = 48). The IG participated in structured plyometric-based game active breaks for 7–10 minutes daily, for six consecutive weeks. The CG resumed their regular daily school routine. FMS were assessed with the Canadian Agility Movement Skills Assessment test, and muscular fitness with the standing long jump (SLJ), countermovement jump (CMJ), and seated medicine ball chest throw tests. The Self-Perception Profile for Children and the Teacher’s Rating Scale of Child’s Actual Behaviour assessed student self-perception and teacher’s perception of student actual behaviour, respectively. A significant (*p* < 0.01) interaction group by time was observed, with greater improvements in the IG compared to the CG in FMS (%diff = 13.11, ηp^2^ = 0.12), SLJ (%diff = 6.67, ηp^2^ = 0.02), seated medicine ball chest throw (%diff = 4.69, ηp^2^ = 0.08), student social self-perception (%diff = 9.31, ηp^2^ = 0.10), student scholastic self-perception (%diff = 7.27, ηp^2^ = 0.10), and teacher perception of student social competence (%diff = 8.31, ηp^2^ = 0.05). No difference (*p* > 0.05) was found in other variables. Integrating plyometric-based structured game active breaks into primary school settings evidenced improvement in FMS, muscular fitness, student self-perception, and teacher’s rating of student actual behaviour.”

This has been corrected to:

“This study examined the effects of plyometric-based structured game active breaks on fundamental movement skills (FMS), muscular fitness, student self-perception, and teacher’s rating of actual behaviour in Grade 3 and 4 students. Primary school children aged 8–10 years old, from four classes, were cluster-randomly assigned to an intervention group (IG) (*n* = 54) or a control group (CG) (*n* = 48). The IG participated in structured plyometric-based game active breaks for 7–10 minutes daily, for six consecutive weeks. The CG resumed their regular daily school routine. FMS were assessed with the Canadian Agility Movement Skills Assessment test, and muscular fitness with the standing long jump (SLJ), countermovement jump (CMJ), and seated medicine ball chest throw tests. The Self-Perception Profile for Children and the Teacher’s Rating Scale of Child’s Actual Behaviour assessed student self-perception and teacher’s perception of student actual behaviour, respectively. A significant (*p* < 0.01) interaction group by time was observed, with greater improvements in the IG compared to the CG in FMS (%diff = 13.11, ηp^2^ = 0.12), SLJ (%diff = 6.67, ηp^2^ = 0.16), seated medicine ball chest throw (%diff = 4.69, ηp^2^ = 0.08), student social self- perception (%diff = 9.31, ηp^2^ = 0.10), student scholastic self-perception (%diff = 7.27, ηp^2^ = 0.10), and teacher perception of student social competence (%diff = 8.31, ηp^2^ = 0.05). No difference (*p* > 0.05) was found in other variables. Integrating plyometric-based structured game active breaks into primary school settings evidenced improvement in FMS, muscular fitness, student self-perception, and teacher’s rating of student actual behaviour.”

